# A new scale for the evaluation of clinical practice guidelines applicability: development and appraisal

**DOI:** 10.1186/s13012-018-0746-5

**Published:** 2018-04-25

**Authors:** Hui Li, Runsheng Xie, Yangyang Wang, Xiuli Xie, Jingwen Deng, Chuanjian Lu

**Affiliations:** 1 Department of Standardization of Chinese Medicine, The Second Affiliated Hospital of Guangzhou University of Chinese Medicine, Guangzhou, Guangdong China; 2 Department of Standardization of Traditional Chinese Medicine, Guangdong Provincial Hospital of Chinese Medicine, Guangzhou, Guangdong China; 3 Engineering and Technology Research Center of Standardization of Traditional Chinese Medicine, Guangzhou, Guangdong China

**Keywords:** Applicability, Clinical practice guideline (CPG), Scale evaluation

## Abstract

**Background:**

This study aimed to develop the clinical practice guidelines applicability evaluation (CPGAE-V1.0) scale and to evaluate its validity and reliability.

**Methods:**

One hundred fifty assessors were invited to rate two rounds of importance scoring of the applicability indicators by using the 5-point Likert scale. Approved indicators formed the CPGAE-V1.0 scale, consisting of 19 items, arranged into 4 domains. We enrolled eligible clinicians from 8 institutions to evaluate 9 clinical practice guidelines using the CPGAE-V1.0 scale. Content validity, construct validity, internal reliability, intra-rater reliability, and responsiveness were analyzed.

**Results:**

A total of 220 clinicians participated, and the response rate was 98.6% (217/220). The CPGAE-V1.0 scale had favorable content validity. The four-factor model produced acceptable fit indices. The scale had an excellent internal consistency and item discrimination. It could identify the degree of applicability of the different dimensions between different guidelines. In all domains, 77.8% (7/9) of CPGs in the minimum-scoring domain were concentrated in the “coordination of support” domain.

**Conclusions:**

The CPGAE-V1.0 scale is a valid and reliable instrument for measuring the applicability of CPG.

**Electronic supplementary material:**

The online version of this article (10.1186/s13012-018-0746-5) contains supplementary material, which is available to authorized users.

## Background

Clinical practice guidelines (CPGs) are playing an increasing role in the development of evidence-based health care, translating the best evidence into best practice principles [1]. Based on the specific clinical circumstances, CPGs can help practitioner and patient make decisions, thereby improving and ensuring medical quality [2, 3]. The aspects of CPG evaluation can be sorted into two parts: development and application. Currently, more than 20 kinds of tools have been adopted to assess and validate CPGs worldwide, and they are designed based on the developer’s perspective [4]. Most of them are a scientific and comprehensive evaluation of CPGs, with a focus on the methodology, the collection of evidence, the reliability of the sources used in the development of the guidelines, etc. However, these tools lack pertinence in evaluating the applicability of CPGs, although these tools are involved in this area. For instance, in the AGREE, AGREE II, and Cluzeau instruments, only three to five items are about the applicability of the scale [5–7]. By using the AGREE instrument, a previous study has found several shortcomings in the applicability of the first batch of Chinese evidence-based CPGs in Traditional Chinese Medicine (TCM) [8]. The study showed that the average score for applicability (27.09%) was the lowest of the six domains because these CPGs failed to sufficiently consider applicability in guideline development. Another study had a similar finding (i.e., the applicability domain not only had the lowest average score but also had the lowest intra-class correlation coefficient (ICC) value) and suggested that experts should focus on improving the applicability of guidelines in the future [9].

In fact, the applicability of a CPG is affected not only by its methodological quality but also by the external environment and conditions in which the CPG is used. For example, in a medical institution, it is necessary to consider whether it meets the requirements of the technology, equipment, staff, laws, and regulations when applying a guideline. From the user’s perspective, applicability evaluation is more concerned with the applicability of CPGs to clinical practice, which obviously differs from their scientific evaluation. Therefore, we believe that the applicability evaluation should include a guideline’s internal characteristics, its external environment, and the interrelationships between them.

With the growing number of CPGs, practitioners may be confused about whether the guideline is suitable according to their current situation. Thus, the present study aimed to develop the clinical practice guidelines applicability evaluation (CPGAE-V1.0) scale to help determine the applicability of CPGs and to evaluate its validity and reliability.

## Methods

### Development of the CPGAE-V1.0 scale

In the first phase, we formed a workgroup of experts in methodologies, hospital administrators, and clinicians. The workgroup members extensively consulted domestic and foreign literatures and related scales and then established 30 items. In addition, 15 self-made items were created after we invited 12 consultants (methodological experts, hospital managers, clinicians) for qualitative interviews. Finally, a total of 45 items were included. In the second phase, 150 assessors (methodological experts and clinicians) were invited to score each item using the 5-point Likert scale vary from 1 (“least important”) to 5 (“very important”). Based on the results of the importance score, approximately 60% of these items were further adjusted or deleted, and then the second-round scoring checklists for 19 items were sent to the same assessors.

### The CPGAE-V1.0 scale content and scoring method

According to the second-round scoring results, the original CPGAE-V1.0 scale was developed, which consisted of 19 items across four domains:(1) technical level, 4 items; (2) coordination of support, 2 items; (3) structure and content, 9 items; (4) the role of the guideline, 4 items (Additional files 1 and 2). A four-point response scale was used to score each item of the CPGAE-V1.0 scale from 1 to 4 (very poor, poor, better, and very good). Supplementary explanations of each item were displayed in the scale to help understand the issues and concepts. The appraisers could list the reasons for their scores in the comment box detailed below each item. The standardized score of each domain (SDS) was calculated as follows [6]:

$$ \mathrm{SDS}=\frac{\mathrm{Observed}\ \mathrm{score}-\mathrm{Minimum}\ \mathrm{possible}\ \mathrm{score}}{\mathrm{Maximum}\ \mathrm{possible}\ \mathrm{score}-\mathrm{Minimum}\ \mathrm{possible}\ \mathrm{score}}\times 100\% $$

In the above formula, observed score = overall domain scores of all the appraisers; minimum possible score = 1 (very poor) × No. of items within a domain × No. of appraisers; maximum possible score = 4 (very good) × No. of items within a domain × No. of appraisers. The overall applicability score of the CPG was also computed in the same standardized method. Higher scores indicated better applicability of the CPG.

### CPGAE-V1.0 investigation

In this phase, from November 2012 to February 2013, the applicability of nine CPGs was evaluated by eight TCM standard research and promotion base construction units, which were located in Guangzhou, Shanghai, Hangzhou, Nanjing, Shenzhen, Fujian, and Qingyuan. These CPGs were issued by the China Association of Chinese Medicine (CACM) in 2008, including menopausal syndrome (MS), chest stuffiness and pains (CSP), exogenous fever, colds, stroke, menstruation, chronic renal failure, transient ischemic attack, and eczema. MS and CSP were key diseases in this study. Eligible clinicians in each participating unit were invited to complete the CPGAE-V1.0 scale to evaluate one of the abovementioned CPGs. Specifically, the evaluator had to meet the following requirements: (1) the evaluator had the relevant professional knowledge involved in the evaluation guideline; (2) the evaluator was not the developer of the evaluation guideline; (3) the evaluator agreed to participate voluntarily in the study. The trained investigators issued and collected the questionnaire strictly in accordance with the survey manual. After we verified the data and filled out the quality control table, relevant documents were sent to the Guangdong Provincial Hospital of Chinese Medicine. The sample size of CPGAE-V1.0 investigation for each key disease was considered at least 50, and for the other diseases, it was considered at least 10. In reality, the effective number of participants in the evaluation survey was 217, and the ratio of sample size to items number was higher than 5:1. To evaluate the intra-rater reliability, one of the participating units had its evaluators re-scored 2 weeks later.

### Statistical analysis

The demographic characteristics of the sample are described. The CPGAE-V1.0 scale was evaluated primarily by validity and reliability analysis. Validity analysis included content validity and construct validity. According to the expert importance score, the content validity index (CVI) was calculated to reflect the magnitude of the content validity, that is, the proportion of items on the 5-point Likert scale that achieved a rating of 3, 4 or 5 within all the assessors. Moreover, we used Bartlett’s test of sphericity and the Kaiser-Meyer-Olkin (KMO) measure of sampling adequacy to examine the appropriateness of the sample size for conducting confirmatory factor analysis (CFA). A CFA model was constructed to analyze the construct validity of the CPGAE-V1.0 scale. The acceptable values of CFA model fit indices are shown in Table 1 [10–17]. Average variance extracted (AVE) was calculated as a test of discriminant validity. Reliability analysis included internal reliability and external reliability. The internal consistency of the total scale, the domains, and the score of the items were evaluated using Cronbach’s alpha coefficient. The scale items were divided into two halves by the odd and even numbers of the items, and the consistency of these two parts’ scores was calculated. The intra-class correlation coefficient (ICC) was used with a two-way random effects model to evaluate the size of the external reliability within each domain and overall score. Responsiveness analysis was intended to reflect the sensitivity of the scale for changes in the characteristics of different CPGs by calculating and comparing the various domains and overall score. We calculated the floor and ceiling effects as the percentage of the participants who had the minimum and maximum scores in each domain, for each item, and overall. Floor or ceiling effects were considered to be present when ≥ 15% of the respondents achieved the minimum or maximum possible score [18]. Missing data were dealt with in the following ways: (1) When more than 20% of items of the questionnaire were missing (that is, the number of missing items was ≥ 4), it was considered invalid and was excluded; (2) when less than 20% of items of the questionnaire were missing (that is, the number of missing items was between 1 and 3), the missing item’s score was set as the average score of the answered items.

**Table 1:** Model fit indices summary

Indices	One-factor model	Four-factor model	Acceptable values
*χ* ^2^	583.34	325.88	
*p* value	< 0.01	< 0.01	> 0.05 [10]
*χ*^2^/df (normed chi-square, NC)	3.84	2.33	< 3.00 [11]
Root mean square error of approximation (RMSEA)	0.12	0.08	< 0.08 [12]
Goodness of fit index (GFI)	0.77	0.86	≥ 0.90 [13]
Adjusted goodness of fit index (AGFI)	0.72	0.81	≥ 0.80 [14]
Normed fit index (NFI)	0.81	0.90	≥ 0.90 [13]
Incremental fit index (IFI)	0.86	0.94	≥ 0.90 [15]
Non-normed fit index (NNFI)	0.84	0.92	≥ 0.90 [16]
Comparative fit index (CFI)	0.85	0.94	≥ 0.90 [17]

In this study, EpiData Version 3.1 (The EpiData Association, Odense, Denmark) was used to build the database and SPSS Version 17.0 (SPSS Inc., Chicago, IL, USA) software was used to process and analyze the data. The CFA model was constructed by IBM® SPSS® Amos ™ 21.0 and confirmatory factor analysis was performed.

## Results

### Characteristics of the sample

A total of 220 clinicians were enrolled in the survey and all completed the survey scale within 20 min. After eliminating three surveys with a proportion of missing items more than 20%, the response rate was 98.6% (217/220). Among the 217 respondents, 96 (44.2%) were chief physicians or associate chief physicians and 121 (55.8%) were resident physicians or attending physicians. The median professional experience was 8 years (IQR 3–16 years).

### Validity study

#### Content validity

Results from the importance scoring by 150 experts showed that the CVI of each item and domain ranged from 0.89 to 0.99 and 0.94 to 0.99, respectively, indicating that the appraisers found the CPGAE-V1.0 scale useful to evaluate CPG and the item and domain settings were satisfactory.

### Construct validity

Bartlett’s test indicated a strong correlation between variables and that we should reject the null hypothesis (chi-square = 3015.72, *p* < 0.001), which showed that factor analysis was appropriate. The KMO statistic of 0.94 (closed to 1.0) reflected that the sum of the correlations was large compared to the sum of the partial correlations, indicating a good fit for factor analysis and adequate sampling in the study.

**Fig. 1:**
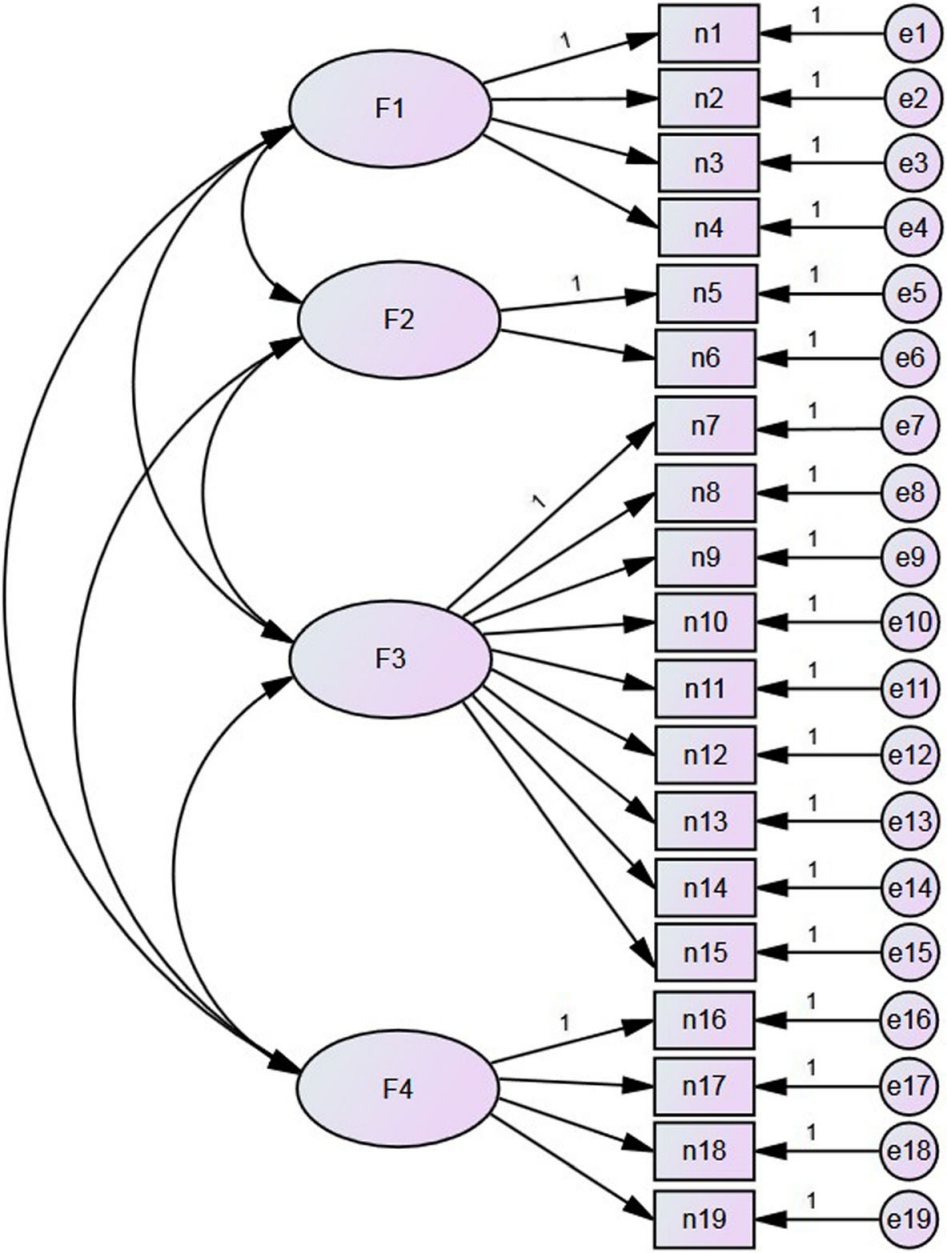
The original proposed four-factor model of the CPGAE-V1.0 scale

**Fig. 2:**
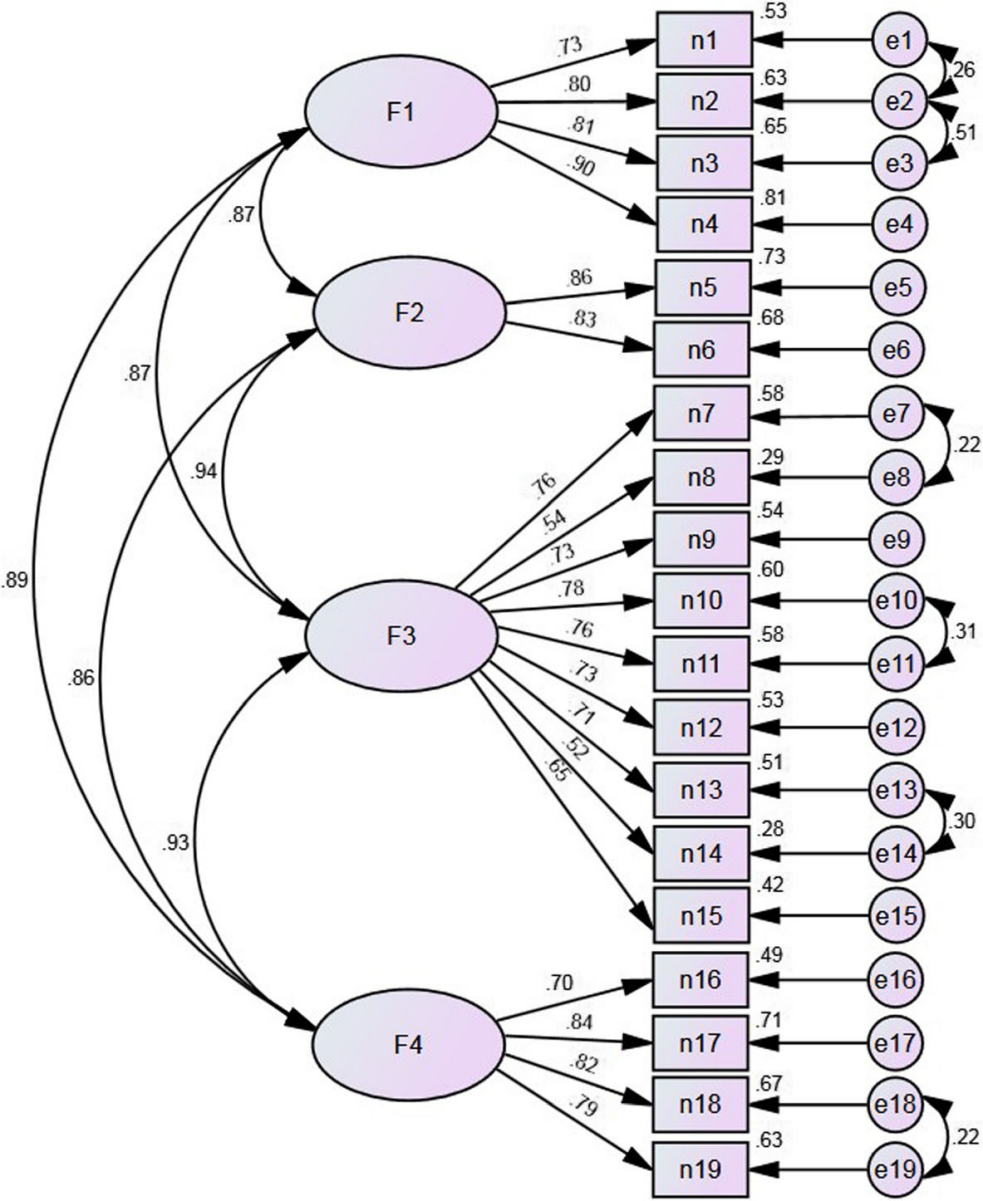
Confirmatory factor analysis of the modified four-factor model of the CPGAE-V1.0 scale (standardized parameter estimates)

A one-factor model was initially implemented, but most of the fit indices were below acceptable thresholds (Table 1). We constructed a four-factor model consisting of a total of 19 items across four domains from CPGAE-V1.0 scale (Fig. 1). After adjusting the covariance relationship between the measurement indicators (items), the model produced acceptable fit indices as shown in Table 1. The CFA results indicated that the best-fitting model was the four-factor solution. Specifically, the normed chi-square was lower than the threshold value of 3, and the AFGI was 0.81, which indicated adequate fit [11, 14]. Furthermore, Fig. 2 and Table 2 show that there was valid evidence of CFA. No items had a factor loading ≤ 0.50 or ≥ 0.95, which conformed to the model recognition rules. Only two items had a factor loading ≤ 0.60 and communality ≤ 0.40: item 8 and item 14.

**Table 2:** Validity evidence

Domain and items	Factor loading	Communality	Measurement error	Average variance extracted	
F1 Technical level				0.66	
n1	0.73	0.53	0.47		
n2	0.80	0.64	0.36		
n3	0.81	0.65	0.35		
n4	0.90	0.81	0.19		
F2 Coordination of support				0.71	
n5	0.86	0.74	0.26		
n6	0.83	0.68	0.32		
F3 Structure and content				0.48	
n7	0.76	0.58	0.42		
n8	0.54	0.29	0.71		
n9	0.73	0.54	0.46		
n10	0.78	0.60	0.40		
n11	0.76	0.58	0.42		
n12	0.73	0.53	0.47		
n13	0.71	0.51	0.49		
n14	0.53	0.28	0.72		
n15	0.65	0.42	0.58		
F4 The role of the guideline				0.63	
n16	0.70	0.49	0.51		
n17	0.84	0.71	0.29		
n18	0.82	0.67	0.33		
n19	0.80	0.63	0.37		

#### Discriminant validity

As shown in Table 2, the AVE of F1, F2, F3, and F4 was 0.66, 0.71, 0.48, and 0.63, respectively. These results suggested that the items in the F3 domain could be further modified to improve the discriminant validity because the AVE of the F3 domain was lower than 0.50.

### Reliability study

#### Internal consistency

For the CPGAE-V1.0 scale, Cronbach’s alpha coefficient was higher than 0.90 and Guttman’s split-half coefficient was 0.96, demonstrating an almost perfect consistency (Table 3). The coefficient of each domain presented acceptable internal consistency (both greater than 0.80). After deleting the domains one by one, the overall reliability of the scale did not increase. Table 3 also shows the correlation between the four domains and the overall scale. With correlation coefficients ranging from 0.83 to 0.95, the domains tended to highly positively correlate with the overall scale. The inter-correlations per domain ranged from 0.64 to 0.84.

**Table 3:** Domain-to-total score correlations (Spearman rank) and the inter-correlations per domain

Domains	Items	*r*_s_	Cronbach’s alpha	Cronbach’s alpha (if domain deleted)	*r*_F1_	*r*_F2_	*r*_F3_	*r*_F4_
F1 Technical level	4	0.85^*^	0.90	0.94	1.00			
F2 Coordination of support	2	0.83^*^	0.82	0.95	0.64^*^	1.00		
F3 Structure and content	9	0.95^*^	0.89	0.94	0.72^*^	0.76^*^	1.00	
F4 The role of the guideline	4	0.93^*^	0.87	0.94	0.74^*^	0.75^*^	0.84^*^	1.00
Total scale	19	1.00	0.95	–				

*P < 0.001

As shown in Table 4, the score of each item was positively correlated with the total score of the scale (the Spearman correlation coefficient ranged from 0.52 to 0.81). In addition to items n8 and n14, other item-to-score correlation coefficients were higher than 0.60. After removing the items one by one, overall Cronbach’s alpha coefficients of the scale were ≤ 0.95, indicating that the items had a good discrimination.

**Table 4:** Item-to-total score correlations (Spearman rank) for the CPGAE-V1.0 scale

Item	*r*_s_^*^	Corrected *r*_s_^*^	Cronbach’s alpha (if item deleted)
n1	0.71	0.72	0.95
n2	0.76	0.75	0.95
n3	0.74	0.72	0.95
n4	0.81	0.80	0.95
n5	0.78	0.78	0.95
n6	0.75	0.75	0.95
n7	0.70	0.74	0.95
n8	0.52	0.53	0.95
n9	0.68	0.69	0.95
n10	0.72	0.74	0.95
n11	0.74	0.74	0.95
n12	0.72	0.71	0.95
n13	0.71	0.70	0.95
n14	0.58	0.52	0.95
n15	0.67	0.62	0.95
n16	0.68	0.66	0.95
n17	0.80	0.78	0.95
n18	0.79	0.77	0.95
n19	0.79	0.76	0.95

*P < 0.001

#### Intra-rater (test-retest) reliability

One of the participating units was selected and eight evaluators were re-scored 2 weeks later. The mean of the overall CPGAE-V1.0 scale standardized score was 81.80 (SD = 8.17) in the first appraisal, and the mean of the score was 81.58 (SD = 5.93) in the second time (*t* = 0.098, *p* = 0.925). The total ICC was 0.76 (0.00 to 0.95), and the domains’ ICCs ranged from 0.29 to 0.85. Weighted Cohen’s kappa varied between − 0.5 and 1.0 with 56.6% (86/152) agreement (Additional file 3).

### Responsiveness analysis

In the nine clinical practice guidelines published in 2008, the climacteric syndrome, menostaxis, and transient ischemic attack guideline was at a high applicability levels (close to 90 points), while chest stuffiness and pains had lower applicability (64.68 points). In all domains, the highest SDS of each CPG are not the same, 77.8% (7/9) of CPGs of the minimum-scoring domain were concentrated in the “coordination of support” domain (Table 5).

**Table 5:** The CPGAE-V1.0 domain scores of nine CPGs

CPG	*N*	F1	F2	F3	F4	Overall applicability score
		Technical level	Coordination of support	Structure and content	The role of the guideline	
CS	62	89.38	85.48	88.77	87.63	88.31
CSP	57	66.23	51.46	66.44	65.79	64.68
EF	11	77.88	83.33	79.12	72.73	77.96
CC	12	76.39	75.00	81.17	73.61	77.92
Ap	24	76.04	73.61	81.48	75.69	78.29
Me	10	90.83	85.00	91.11	87.50	89.65
CRF	16	72.40	66.67	71.56	67.71	70.41
TIA	10	89.17	86.67	89.26	91.67	89.47
Ec	15	78.52	74.44	75.80	78.33	76.76

Abbreviations: *CS* climacteric syndrome, *CSP* chest stuffiness and pains, *EF* exogenous fever, *CC* common cold, *Ap* apoplexy, *Me* menostaxis, *CRF* chronic renal failure, *TIA* transient ischemic attack, *Ec* eczema

#### Floor and ceiling effect

There were no floor effects in each domain, for each item, and overall. There were no ceiling effects in overall score (9.7%). Form F1 to F4, the ceiling effects were 34.6, 32.7, 12.0, and 23.5%, respectively.

## Discussion

The present paper describes a multi-staged development process, as well as a validity and reliability study of the CPGAE-V1.0 scale. This is a reliable and effective assessment tool designed to provide a framework for measuring the applicability of clinical practice guidelines to help users understand the inadequacies of the guidelines and make choices.

The factor analysis results confirmed our conceptual framework of applicability, lending support to the assumption that the applicability of clinical guidelines is composed of four distinct domains, each domain assessed by its key items. Benefitting from the importance scoring of 150 assessors in two rounds, the valuable and approbatory items and domains were screened out. Furthermore, the measurement variables in CFA could be interpreted to a higher degree because each item exhibited sufficient factor loading (both greater than 0.50). However, the F3 domain (structure and content) indicated that slightly weaker discriminant validity may be weakened by the lower AVE. Within the F3 domain, the item-to-total score correlation coefficient of n8 and n14 were lower than other items, and the communality of items n8 and n14 were lower than the suggested minimum of 0.40. To improve the explanatory power of the F3 domain, its items could be further modified, especially items n7 and n8. Although the four-factor model had favorable construct validity, we do not know the relative contribution of each domain to the overall applicability of a guideline. A similar situation exists in other assessment instruments, and we need to explore solutions in the future [6].

Our reliability analysis results demonstrated that the CPGAE-V1.0 scale had an excellent internal consistency and item discrimination. In general, all the domains and the total score reached the recommended minimum of 0.70 for Cronbach’s alpha coefficient, meaning they should be considered acceptable [18]. As shown in Table 3, Cronbach’s alpha coefficient of the CPGAE-V1.0 scale was satisfactory (≥ 0.95). These items were correlated with the measurement purpose of the scale, and the overall reliability of the scale did not increase if any item was excluded. However, poor intra-rater reliability was found, and the possible reasons are follows. First, poor retest reliability is related to the setting of the range and pre-evaluation training. On rechecking the original record, more than 95% of raters scored each item as “better” or “very good”, which were in the same direction but different classification. This reflected that the grading of the scale was not clear enough and pre-evaluation training did not fully clarify the distinction between levels. Second, an insufficient retest sample size resulted in increased sampling error and weakened the reliability and stability.

Previous studies have found several shortcomings in the applicability of CPGs of Traditional Chinese Medicine and suggest that we should pay special attention to improve this area in the future [8, 9]. At the time of this study, nine TCM clinical practice guidelines had been released for almost 5 years. Our evaluation results indicate that the applicability of some guidelines is still high (i.e., the CS and TIA guidelines, which earned close to 90 points), while the individual guidelines are relatively low (i.e., CSP, 64.68 points). These data suggest that developers should consider revising the guidelines to improve the level of applicability. In all domains, most CPGs (77.8%) in the minimum-scoring domain were concentrated in the “coordination of support” domain. According to the composition of the domain, the data indicate that these guidelines lack coordination to the other relevant standards or guidelines in interrelated content. Meanwhile, the relevant medical resources (such as medical technology and operating room), which should be supported in the implementation, are insufficient. Previous studies suggested users evaluate the quality of CPGs before adopting them [6]. However, some authors have shown that the methodological quality of CPGs may not necessarily equal the validity of recommendations, which confuses practitioners when deciding on the appropriate guideline [19, 20]. From a practitioner’s perspective, they are more concerned with whether the guideline is applicable for clinical practice in the situation they are facing. Under these circumstances, we recommend using the CPGAE-V1.0 scale in helping to understand the applicability of CPGs before adopting them. In this way, they can assess the applicability of CPGs to clinical practice through the applicability evaluation.

Taking into account the feasibility of the practical application, each item of the CPGAE-V1.0 scale listed relevant supplementary explanation for the appraisers to understand the issues and concepts involved in the item. As completing the scale does not involve any complex calculations, the effective response rate of participation was relatively high. However, there are some limitations in the current study. First, participation in this study was limited to clinicians who played a major role in the medical decision-making process, but we did not survey other medical staff who also participate in medical decision-making, such as nurses. Second, a 4-point response scale was used to score each item of the CPGAE-V1.0 scale, referring to the AGREE instrument, while some studies considered that a 7-point response scale may have been more in compliance with methodological requirements and instrument reliability [7, 21]. However, a 7-point response scale may be more difficult and take more time to finish [22]. Finally, the low sample size of intra-rater reliability is another disadvantage. According to the COSMIN checklist, this small sample size (< 30) included in the intra-rater reliability analysis is poor [23]. An inadequate sample size will weaken the reliability and stability of the retest results (i.e., lower kappa values). When other researchers use this tool in the future, they could refer to the COSMIN checklist and involve more than 100 raters in the retest to improve the reliability of the results. This is also the focus of our next version. We grant that the development of this first version of a clinical practice guidelines applicability evaluation scale was not perfect, but we hoped that this report will inspire other researchers in this field to conduct similar studies.

## Conclusions

The applicability evaluation of clinical practice guidelines is a creative and challenging endeavor. Our findings indicate that the CPGAE-V1.0 scale is a valid and reliable instrument for measuring the applicability of CPGs. This scale can be used conveniently to evaluate the applicability of CPGs in practical applications and to find their deficiencies to promote the application and improvement of CPGs.

## Additional files

Additional file 1: CPGAE-V1.0 scale (Chinese version). (PDF 360 kb)

Additional file 2: CPGAE-V1.0 scale (English version). (PDF 156 kb)

Additional file 3: **Table S1.** Weighted Cohen’s kappa of each item. (DOCX 32 kb)

## Abbreviations

AGFI: Adjusted goodness of fit index
Ap: Apoplexy
AVE: Average variance extracted
CACM: China Association of Chinese Medicine
CC: Common cold
CFA: Confirmatory factor analysis
CFI: Comparative fit index
CPG: Clinical practice guideline
CPGAE-V1.0: Clinical practice guidelines applicability evaluation
CRF: Chronic renal failure
CS: Climacteric syndrome
CSP: Chest stuffiness and pains
CVI: Content validity index
Ec: Eczema
EF: Exogenous fever
GFI: Goodness of fit index
ICC: Intra-class correlation coefficient
IFI: Incremental fit index
KMO: Kaiser-Meyer-Olkin
Me: Menostaxis
MS: Menopausal syndrome
NC: Normed chi-square
NFI: Normed fit index
NNFI: Non-normed fit index
RMSEA: Root mean square error of approximation
SDS: The standardized score of each domain
TCM: Traditional Chinese Medicine
TIA: Transient ischemic attack
