# Supplementary material for: A new scale for the evaluation of clinical practice guidelines applicability: development and appraisal
Source: Implement Sci. 2018 Apr 25;13:61. doi: 10.1186/s13012-018-0746-5 (PMC5918771; doi:10.1186/s13012-018-0746-5)
Supplement: Supplementary file 1 — CPGAE-V1.0 scale (Chinese version). (PDF 360 kb) [file 13012_2018_746_MOESM1_ESM.pdf]

编号□□□□

临床实践指南适用性评价量表

指南名称：______________________________

单　　位：_______________________（盖章）

姓　　名：______________________________

专　　业：______________________________

职　　称：正高□　副高□　中级□　初级□

从事本专业时间：______________年

填表时间：______________________________

版块一：技术水平

1．与我国卫生医疗水平相比的适应性

很好	4	3	2	1	很差

意见

2．与本地区医疗水平相比的适应性。

很好	4	3	2	1	很差

意见

3．与本单位医疗水平相比的适应性。

很好	4	3	2	1	很差

意见

4．与其他相关诊疗方案水平比较。

很好	4	3	2	1	很差

意见

1.与我国卫生医疗水平相比的适应性

考察指南所规定的技术水平与当前我国在该领域的主流或平均的研究水平、服务水平、技术水平、管理水平等相比是否适应。

2.与本地区卫生医疗水平相比的适应性

考察指南所规定的技术水平与当前本地区在该领域的主流或平均的研究水平、服务水平、技术水平、管理水平等相比是否适应。

3. 与本单位卫生医疗水平相比的适应性

考察指南所规定的技术水平与当前本单位在该领域的主流或平均的研究水平、服务水平、技术水平、管理水平等相比是否适应。

4. 与其他相关诊疗方案水平比较

考察指南所规定的技术水平与该领域相关诊疗方案的主流或平均的研究水平、服务水平、技术水平、管理水平等相比是否适应。

版块二：协调配套性

5. 与相关标准（指南）的内容协调配套性。

很好	4	3	2	1	很差

意见

6. 得到多学科（含医技/手术室等）的协调配合。

很好	4	3	2	1	很差

意见

版块三：结构和内容

7. 指南适用范围明确。

很好	4	3	2	1	很差

意见

8. 指南的诊断要点准确。

很好	4	3	2	1	很差

意见

5．与相关标准（指南）的内容协调配套性。

指被评价的指南与相关标准或者相关的指南在内容上的相互关联、相互协调，能够配套使用。

6．得到多学科（含医技/手术室等）的参与和协调配合

指被评价的指南在实施时得到多学科（如医技、手术室等）的关注、参与和协调配合。

7．指南适用范围明确

指南适用范围应明确表明指南适用的对象和所涉及的各个方面，由此指明指南或其特定部分的适用界限。必要时.可指出指南不适用的界限。

8．指南的诊断要点准确

指南的诊断要点应对该病种的主要诊断依据和诊断方法作简要的陈述，提出主要诊断依据，包括主要的和（或）相对特异性的症状、体征等。叙述时应主次分明，先写作为诊断的主要依据的指标再写次要的或参考指标。

9. 指南理化检查合理。

很好	4	3	2	1	很差

意见

10. 指南结构完整、合理。

很好	4	3	2	1	很差

意见

11. 指南内容完整、合理。

很	4	3	2	1	好 很差

意见

12. 指南的内容清晰明了。

很好	4	3	2	1	很差

意见

9. 指南理化检查合理。

指南中涉及的理化检查指标均有其相应的有效可行的检测方法，检测方法应结合实际情况避免过于复杂或成本太高。

10. 指南的结构完整、合理。

从指南的结构合理性和内容完整性、或者便于医务人员使用指南等角度出发进行评价。

11. 指南的内容完整、合理。

重点考察指南的技术内容上存在的问题。依照问题的严重程度，分为下列情况:

（1）基本无存在问题，内容科学合理。

（2）存在需要细微改动或补充的地方，这类问题可以通过指南修改通知单的形式进行修改。如：需要对指南中已达成一致的技术条款作适当的补充或修改，但不足以影响到指南的主要技术内容。

（3）存在一些问题，如：指南的内容不够全面需要补充；随着医学的发展，指南的个别技术指标需要修订；指南中的技术指标没有量化；指南的技术内容过于繁琐。

（4）存在严重问题，如：指南与法律法规或强制性标准相抵触。

12. 指南的内容清晰明了。

指指南的内容表达清楚明晰，容易被大多数临床医疗工作者理解。

13. 指南的技术内容之间互相配套到位。

很好	4	3	2	1	很差

意见

14. 指南的内容之间无互相矛盾。

很好	4	3	2	1	很差

意见

15. 指南的可扩展性。

很好	4	3	2	1	很差

意见

版块四：指南的作用

16. 指南临床应用的简便性。

很好	4	3	2	1	很差

意见

13、指南的技术内容之间互相配套到位。

指指南各部分技术内容相互衔接和有机配套。指南内容有重复也可以判定为不协调，不配套。

14、指南的内容之间无互相矛盾。

指指南各部分的内容没有出现不一致或者前后不协调的情况。

15、指南的可扩展性

随着医疗技术的发展，指南的个别技术指标需要修订。指南的可扩展性是指指南在内容、结构上为未来不断发展变化的医疗知识更新提供了可扩展的余地。

16.指南临床应用的简便性。

主要考察指南诊疗方案的临床可操作性，是否方便可行。

17. 指南合理利用医疗资源。

很好	4	3	2	1	很差

意见

18. 指南在规范医疗管理、保障医疗服务质量方面的作用。

很好	4	3	2	1	很差

意见

19. 指南在提高医疗技术水平方面的作用。

很好	4	3	2	1	很差

意见

17.指南合理利用医疗资源

指南的实施有助于合理利用现有的医疗投入、资源、人才配备，减少无效劳动。

18.指南在规范医疗管理、保障医疗服务质量方面的作用

是指指南中的规范性内容是否优于本单位现行的管理水平，采用新指南能否进一步提高医疗服务管理和质量水平，及其具体作用分析。

19.指南在提高医疗技术水平方面的作用

是指指南的技术内容是否有助于提高该病种目前的医疗技术水平和技术特色。
