# Supplementary material for: A new scale for the evaluation of clinical practice guidelines applicability: development and appraisal
Source: Implement Sci. 2018 Apr 25;13:61. doi: 10.1186/s13012-018-0746-5 (PMC5918771; doi:10.1186/s13012-018-0746-5)
Supplement: Supplementary file 2 — CPGAE-V1.0 scale (English version). (PDF 156 kb) [file 13012_2018_746_MOESM2_ESM.pdf]

ID □□□□

Clinical Practice Guidelines Applicability Evaluation Scale

Guideline name: ______________________________

Institution: __________________________________

Name: ______________________________________

Major: ______________________________________

Professional title: _____________________________

Engaged in the professional time: _____________

Date: _______________________________________

Domain 1: Technical level

1. Compared to the country health care level.

Very good	4	3	2	1	Very poor

Comment

2. Compared to the local health care level.

Very good	4	3	2	1	Very poor

Comment

3. Compared to the unit health care level.

Very good	4	3	2	1	Very poor

Comment

4. Compared to other related clinical and diagnosis programs.

Very good	4	3	2	1	Very poor

Comment

Domain 2: Coordination of support

5. Coordinate with the contents of the relevant standards or guidelines.

Very good	4	3	2	1	Very poor

Comment

6. Coordinate with multidisciplinary.

Very good	4	3	2	1	Very poor

Comment

Domain 3: Structure and content

7. The scope of application is clear.

Very good	4	3	2	1	Very poor

Comment

8. The diagnostic point is accurate.

Very good	4	3	2	1	Very poor

Comment

9. The physico-chemical examination is reasonable.

Very good	4	3	2	1	Very poor

Comment

10. The structure is complete and reasonable

Very good	4	3	2	1	Very poor

Comment

11. The content is complete and reasonable.

Very good	4	3	2	1	Very poor

Comment

12. The content is clear.

Very good	4	3	2	1	Very poor

Comment

13. Technical contents support each other.

Very good	4	3	2	1	Very poor

Comment

14. There is no contradiction between the contents.

Very good	4	3	2	1	Very poor

Comment

15. The extensibility of the guideline

Very good	4	3	2	1	Very poor

Comment

Domain 4: The role of the guideline

16. The convenience of clinical application.

Very good	4	3	2	1	Very poor

Comment

17. Rational use of medical resources.

Very good	4	3	2	1	Very poor

Comment

18. The role of regulating medical management and guaranteeing medical service quality.

Very good	4	3	2	1	Very poor

Comment

19. The role of improving medical technology level.

Very good	4	3	2	1	Very poor

Comment

Explanations

Item 1, item 2, item 3 and item 4. The technical level stipulated in the guideline should be suitable for the current mainstream or average level of research, service, technical and management of the country/local/unit/ related health care level in this field.

Item 5. The guideline should be coordinated with the contents of the relevant standards or guidelines.

Item 6. The guideline should be coordinated with multidisciplinary implementation.

Item 7. The guideline should clearly indicate the scope of application and the population(s) that is affected by the recommendation(s).

Item 8. The guideline should accurately describe the main diagnosis and diagnostic methods of the disease for a brief statement.

Item 9. The physical and chemical examination indicators involved in the guidelines should have effective and feasible detection methods.

Item 10 and item 11. The structure and content of the guideline should be complete and reasonable for the medical staff to use. The problems in the technical content can be divided into the following situations: ①The content is scientific and reasonable. ②The content needs minor changes or additions. ③The content has some problems. ④The content has serious problems (e.g., the guideline conflicts with laws, regulations or mandatory standard).

Item 12. The content of the guideline should be clear and easy to understand by most clinicians.

Item 13 and item 14. Technical contents of the guideline should be support and coordinate with each other, without any inconsistency.

Item 15. With the development of medical technology, some technical indicators of the guideline need to be revised. The extensibility of the guideline means that the guideline should have certain space for updating its content and structure in the future.

Item 16. Clinical diagnosis and treatment program of guideline should be feasible and convenient.

Item 17. The implementation of guideline should help to use rational medical resources.

Item 18. The implementation of guideline should further to regulate medical management and guarantee service quality.

Item 19. Technical contents of the guideline should help improve the current medical technology level.
