# Supplementary material for: A new scale for the evaluation of clinical practice guidelines applicability: development and appraisal
Source: Implement Sci. 2018 Apr 25;13:61. doi: 10.1186/s13012-018-0746-5 (PMC5918771; doi:10.1186/s13012-018-0746-5)
Supplement: Supplementary file 3 — Table S1. Weighted Cohen’s kappa of each item. (DOCX 32 kb) [file 13012_2018_746_MOESM3_ESM.docx]

**Table S1** Weighted Cohen's kappa of each item

| Item | Weighted Cohen's kappa(95% CI) |
| --- | --- |
| n1 | 0.3 (-0.1, 0.8) |
| n2 | 0.0 (0.0, 0.0) |
| n3 | 0.0 (-0.6, 0.6) |
| n4 | 0.1 (-0.5, 0.8) |
| n5 | 0.5 (0.0, 1.0) |
| n6 | -0.2 (-0.4, 0.1) |
| n7 | 0.1 (-0.5, 0.8) |
| n8 | 0.3 (-0.2, 0.7) |
| n9 | -0.3 (-0.9, 0.4) |
| n10 | 0.1 (-0.5, 0.8) |
| n11 | 0.0 (0.0, 0.0) |
| n12 | 0.0 (0.0, 0.0) |
| n13 | 0.2 (-0.2, 0.5) |
| n14 | 1.0 (1.0, 1.0) |
| n15 | 0.0 (0.0, 0.0) |
| n16 | -0.5 (-1.0, 0.1) |
| n17 | 0.3 (-0.1, 0.8) |
| n18 | 0.2 (-0.2, 0.5) |
| n19 | 0.1 (-0.1, 0.3) |
| Total | 0.1 (0.0, 0.3) |
